# Association between nighttime sleep duration and cognitive function in middle-aged and older adult patients with multimorbidity: the mediating role of depression

**DOI:** 10.3389/fpubh.2025.1576629

**Published:** 2025-07-09

**Authors:** Ming Jia, Xingyu Liu, Xiuwei Da

**Affiliations:** ^1^Xi’an Innovation College of Yan’an University, Xi’an, China; ^2^Department of Interventional Radiology, Tangdu Hospital, Fourth Military Medical University, Xi’an, China

**Keywords:** multimorbidity, nighttime sleep duration, cognitive function, depression, chronic disease

## Abstract

**Objective:**

To investigate the mediating role of depression in the relationship between nighttime sleep duration and cognitive function among middle-aged and older adult patients with multimorbidity, providing insights for mitigating cognitive decline.

**Methods:**

Utilizing data from the China Health and Retirement Longitudinal Study (CHARLS) (2015 and 2020 waves), 4,210 participants with ≥2 chronic conditions were included. Correlation, regression, and mediation analyses were conducted to examine associations between sleep duration, depression, and cognitive function.

**Results:**

The prevalence of cognitive impairment was 35.7%. Nighttime sleep duration showed a weak positive correlation with cognitive function (*r* = 0.071, *p* < 0.01) and a stronger negative correlation with depression (*r* = −0.251, *p* < 0.01). Depression was negatively correlated with cognitive function (*r* = −0.262, *p* < 0.01). Mediation analysis revealed that depression fully mediated the sleep-cognition relationship [indirect effect: 0.120, 95% CI (0.100–0.141); direct effect nonsignificant].

**Conclusion:**

Depression fully mediates the association between nighttime sleep duration and cognitive function in multimorbidity patients. Interventions targeting sleep hygiene and mental health may synergistically alleviate cognitive decline in this population.

## Introduction

According to the World Health Statistic 2023 (1), the proportion of deaths due to chronic diseases has increased from 61 to 74% globally, which has a serious impact on the health of the population. Chronic diseases have a long duration and complex treatment, and individuals often suffer from multiple chronic diseases at the same time. In 2008, the World Health Organization (WHO) defined multimorbidity as “the simultaneous presence of two or more chronic diseases in the same patient” (2). The study found that 46.5% of Chinese adults were patients with multimorbidity. The prevalence of cognitive impairment was significantly higher in patients with multiple diseases compared to those with a single chronic disease. Cognitive impairment is a common disease in the older adult population, and it is estimated that about 15.6% of people aged 50 years and older suffer from mild cognitive impairment, which progresses to Alzheimer’s disease and dementia over time (3, 4). It not only affects the quality of life of the older adult but also imposes a serious burden on families and society. To address the significant disease burden caused by cognitive impairment, it is crucial to identify the risk factors associated with cognitive impairment (5).

Sleep is an important factor in the health of older adults. Studies have shown an association between changes in sleep parameters and cognitive decline or recovery. Sleep deprivation accelerates neuronal cell loss in the frontal, parietal, and temporal lobes of the brain, affecting synaptic plasticity and neuronal function, which can lead to cognitive impairment and even dementia (6). A cohort study found that patients with sleep disorders (insomnia, rapid eye movement sleep behavior disorder, and excessive daytime sleepiness) had a significantly increased risk of developing dementia compared to patients without sleep disorders (7). Therefore, sleep disorders are considered as possible potential triggers or biomarkers of altered cognitive function, and monitoring sleep quality is expected to serve as a non-pharmacological intervention to assess the risk of future Alzheimer’s disease or to monitor the effectiveness of clinical interventions. In addition, sleep deprivation serves as an important predisposing factor for cardiovascular diseases, inflammation, and metabolic disorders, and patients with multimorbidity are subject to cumulative and synergistic effects developed by multiple diseases, which leads to the impact of individual psychological state in the older adult, which in turn induces anxiety, depression, and other emotions.

Depression is a common psychological disorder, and there is a close symbiotic and causal bidirectional relationship between depression and cognitive impairment. Depression in old age is often accompanied by cognitive decline. Existing research suggests that depression can have complex effects on cognitive function through biological perspectives. Depression leads to an imbalance of neurotransmitters such as 5-hydroxytryptamine and dopamine, a decrease in brain neuroplasticity, and an abnormality of the signal transduction system in the brain, which in turn induces the onset and development of cognitive dysfunction (8). Whereas cognitive impairment is also often accompanied by depression, cognitive decline may lead to decreased self-care, gradual memory loss, limited social interactions, and an increased likelihood of experiencing self-worth denial and helplessness, ultimately leading to depressive symptoms. A systematic review indicated that cognitive decline can be slowed and depressive symptoms improved with cognitive behavioral therapy (9).

Much of the previous research has focused on the independent effects of sleep duration or depression on cognitive functioning and has tended to overlook this joint effect. The mechanisms by which nighttime sleep duration and depression interact to influence cognitive functioning outcomes remain unclear, and research on the potential mediating role of depression in the relationship between nighttime sleep duration and cognitive functioning is notably lacking. Therefore, this study aimed to analyze whether depressive symptoms mediate the relationship between nighttime sleep duration and cognitive functioning, to provide a reference for the prevention and alleviation of cognitive functioning in middle-aged and older adult patients with multimorbidity.

## Materials and methods

### Sample and data collection

The China Health and Retirement Longitudinal Study (CHARLS), a publicly available database accessible via http://charls.Pku.edu.cn/en is a long-term tracking survey project co-sponsored by the Institute of Sociology of the Chinese Academy of Social Sciences and the National School of Development at Peking University. Targeting residents of mainland China, it was conducted in 28 provincial-level units, 150 county-level units, and 450 village-level units in China, with the aim of collecting data on middle-aged and older adult individuals and households in China. The survey covers health status and functioning, family structure, and economic income, making it highly representative. The data collection process included face-to-face interviews conducted by trained interviewers using standardized procedures. Follow-up surveys for the project are conducted every 2 years, and four have been conducted so far, in 2013, 2015, 2018, and 2020. The project is consistent with the principles outlined in the Declaration of Helsinki and was approved by Peking University Institutional Review Board (IRB 00001052-11015). Prior to participation in the investigation, all subjects were voluntarily enrolled and signed a written informed consent form. Confidentiality measures such as anonymization of data were implemented to protect patient privacy.

**Figure 1:**
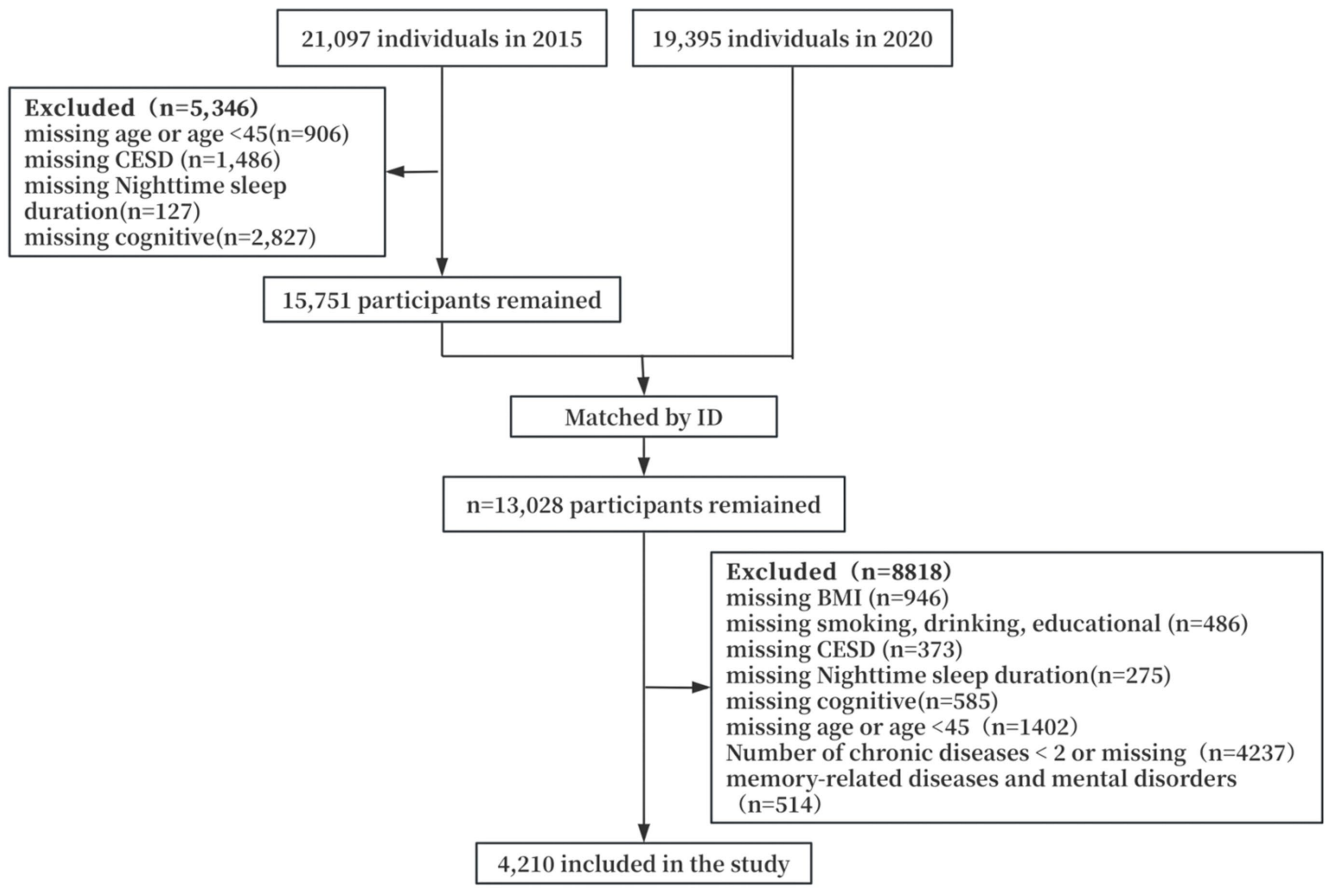
Screening flowchart.

In this study, we analyzed data obtained in 2015 and 2020 and used the 2015 survey as a baseline to finally include 4,210 middle-aged and older adult patients with multimorbidity in the study. Inclusion and exclusion criteria were as follows: (1) Multi-diseased patients: individuals with ≥2 chronic diseases at the same time. Chronic diseases enumerated in this study included hypertension, diabetes mellitus, cancer or malignant tumors, chronic lung diseases (e.g., chronic bronchitis and emphysema), heart disease, stroke, arthritis, dyslipidemia, liver disease, renal disease, gastric or other digestive disorders, asthma, etc.; (2) no missing data on key variables such as nighttime sleep duration, depression scores, cognitive function scores, and so on; (3) exclusion of patients with missing data on sociodemographic characteristics and health-related variables; (4) excluding patients with memory-related diseases and mental disorders. The specific procedure is shown in Figure 1.

## Measures

### Nighttime sleep duration

To assess self-reported hours of nighttime sleep, investigators assessed self-reported hours of nighttime sleep by asking respondents, “In the past month, how many hours of actual sleep did you get at night (the average number of hours of sleep per night)?” (from the 2015 CHARLS follow-up questionnaire, Question ID: DA049) The American Academy of Sleep Medicine recommends 7–9 h of sleep for adults and 7–8 h for older adults (10). In this study, sleep duration of less than 7 h was defined as the short sleep group, 7–8 h as the normal sleep group, and more than 8 h as the long sleep group.

### Depression

Depression was evaluated using the 10-item Center for Epidemiologic Studies Depression Scale (CES-D), which consists of 10 self-assessment questions with 2 positive and 8 negative entries, each with 4 options: “rarely or not at all” was assigned a score of 0, “not too much” was assigned a score of 1, “sometimes or half the time” was assigned a score of 2, “most of the time” assigned a score of 3. Positive entries were required to be scored negatively; a total depression score of ≥10 was characterized as having depressive symptoms, and <10 as having no depressive symptoms.

### Cognitive function

The cognitive functioning measure consists of two components: situational memory and mental state. Situational Memory: After the interviewer read a list of 10 words, the respondent was asked to recall the words (immediate recall). Approximately 10 min later, the respondent is asked to repeat the original word (delayed recall). Mental Status: was assessed through the Telephone Interview for Cognitive Status (TICS) and visual structure. The TICS consisted of date orientation and numeracy, in which date orientation required the participant to answer today’s date (day of the year, month, day of the week, and season) and what day of the week it is; numeracy required the participant to compute the value of a number by subtracting 7 consecutively, starting from 100, up to a maximum of 5 times; 1 point was awarded for each correct answer or computation, for a total score of 10 points for the TICS. Visual constructions consisted of the investigator showing the subject a picture and asking the subject to redraw it, with one point awarded for a correct drawing. Tics and visual constructions were summed to give a total mental status score of 11 points. The sum of the situational memory and mental state scores is the total cognitive functioning score. In this study, cognitive functioning scores below 11 were defined as cognitively impaired and vice versa.

### Covariates

Based on previous studies, socio-demographic characteristics were determined to include, age, sex (male/female), place of residence (rural/urban), education level (illiterate/elementary/junior high/middle/high school and above), and marital status (single/married). Health-related factors included whether or not they smoked, whether or not they drank alcohol, and body mass index (BMI), which is calculated as weight (kg) divided by the square of height (m), and was categorized into four groups according to Chinese standards: underweight (<18.5 kg/m^2^), normal (18.5–23.9 kg/m^2^), overweight (24–27.9 kg/m^2^), and obese (≥28 kg/m^2^).

### Statistical analyses

SPSS25.0 statistical software package was used to analyze the data. Count data were described as frequency and percentage, and count data were described as mean and standard deviation; Pearson’s correlation analysis was used to analyze the relationship between cognitive function, depressive symptoms, and nighttime sleep duration in middle-aged and older adult multimorbidity patients; Model 4 in SPSS macro program PROCESS 4.1 was used to explore the moderating mediating role of depressive symptoms in the relationship between nighttime sleep duration and cognitive function. The mediating effect was tested by the Bootstrap method. The test level *α* = 0.05.

## Result

### Basic characteristics

Of the 4,210 participants, the median age was 61 years (interquartile range: 55–67 years); 2,237 (53.1%) were female, 1973 (46.9%) were male, 3,713 (88.2%) were married, 1,667 (39.6%) illiterates, 2,631 (62.5%) resided in a rural area, 1,865 had 2 chronic diseases (44.3%), 1,579 (37.5%) with depressive symptoms, and 1,503 (35.7%) with cognitive impairment. The results of the univariate analysis showed that the occurrence of cognitive impairment differed statistically significantly (*p* < 0.05) between age, gender, place of residence, education, marital status, number of chronic diseases, smoking, alcohol consumption, BMI, nighttime sleep duration, and depression. Other information is detailed in Table 1.

**Table 1:** Basic characteristics of participants by cognitive function (*n* = 4,210).

Variables	Non-cognitive impairment (*n* = 2707)	Cognitive impairment (*n* = 1503)	Statistic	*p*
Age, mean(SD)	60.00 (53.00, 66.00)	63.00 (58.00, 69.00)	*Z* = −10.78	<0.001
Sex, *n* (%)			*χ*^2^ = 129.26	<0.001
Male	1445 (53.38)	528 (35.13)		
Female	1262 (46.62)	975 (64.87)		
Marital status, *n* (%)			*χ*^2^ = 52.33	<0.001
Single	247 (9.12)	250 (16.63)		
Married	2460 (90.88)	1253 (83.37)		
Educational level, *n* (%)			*χ*^2^ = 882.95	<0.001
Illiterate	644 (23.80)	1023 (68.06)		
Primary school	786 (29.03)	315 (20.96)		
Middle school	804 (29.70)	136 (9.05)		
High school and above	473 (17.47)	29 (1.93)		
Family residence, *n* (%)			*χ*^2^=106.28	<0.001
Urban	1170 (43.22)	408 (17.47)		
Rural	1537 (56.78)	1094 (72.83)		
Number of chronic conditions, *n* (%)			*χ*^2^ = 50.078	<0.001
2	1115 (41.19)	750 (49.90)		
3	725 (26.78)	420 (27.94)		
≥4	867 (32.03)	333 (22.16)		
Drinking, *n* (%)			*χ*^2^ = 32.48	<0.001
No	1359 (50.20)	892 (59.35)		
Yes	1348 (49.80)	611 (40.65)		
Smoking, *n* (%)			*χ*^2^ = 36.33	<0.001
No	1429 (52.79)	938 (62.41)		
Yes	1278 (47.21)	565 (37.59)		
BMI			*χ*^2^ = 43.02	<0.001
Underweight, *n* (%)	95 (3.51)	100 (6.66)		
Normal weight	1118 (41.30)	708 (47.10)		
Overweight	1015 (37.50)	472 (31.40)		
Obesity	479 (17.69)	223 (14.84)		
Nighttime sleep duration, *n* (%)			*χ*^2^ = 23.975	<0.001
Short sleep	1473 (54.40)	868 (57.75)		
Normal sleep	1030 (38.04)	475 (31.60)		
Long sleep	205 (7.56)	160 (10.65)		
Nighttime sleep duration, mean ± SD)	6.35±1.71	6.02±2.19	*t* = 5.06	<0.001
Depression score, mean (SD)	6.00 (3.00, 11.00)	9.00 (5.00, 15.00)	*Z* =14.49	<0.001

*t* = *t*-test; Z = rank sum test; χ^2^ = chi-square test; BMI=Body Mass Index.

### Analysis of the correlation between nighttime sleep duration, depression, and cognitive function in middle-aged and older adult patients with multimorbidity

Nighttime sleep duration was positively correlated with cognitive function (*r* = 0.071, *p* < 0.01). Total depression score was negatively correlated with nighttime sleep duration (*r* = −0.251, *p* < 0.01). The total depression score was negatively correlated with the total cognitive function score (*r* = −0.262, *p* < 0.01). The results are shown in Table 2.

**Table 2:** Correlation coefficients of nighttime sleep duration, depression, and cognitive function in middle-aged and older adult multimorbidity patients.

Variables	Nighttime sleep duration	Depression	Cognitive function
Nighttime sleep duration	1.000		
Depression	−0.251*	1.000	
Cognitive function	0.071*	−0.262*	1.000

*p < 0.01.

In the short-sleep group, there was a significant negative correlation between the duration of nighttime sleep and cognitive function (*r* = −0.202); in the normal-sleep group, there was no significant correlation between the duration of nighttime sleep and cognitive function (*r* = 0.044); and in the long-sleep group, there was a significant positive correlation between the duration of nighttime sleep and cognitive function (*r* = 0.105). The results are shown in Table 3.

**Table 3:** Correlations between nighttime sleep duration and cognitive function in different nighttime sleep duration subgroups.

Nighttime sleep duration subgroups	Sample size (*n*)	Correlation coefficient (*r*)	*p*
Short-sleep group < 7h	2341	−0.202	<0.01
Normal sleep group 7–8h	1505	0.044	>0.05
Long sleep group > 8h	364	0.105	<0.05

### Nighttime sleep duration mediates depression and cognitive functioning in middle-aged and older adult multimorbidity

Regression analysis using nighttime sleep duration as the independent variable and cognitive function as the dependent variable showed that nighttime sleep duration was an influencing factor of cognitive function (*p* < 0.001); using nighttime sleep duration as the independent variable and depression as the dependent variable, the results showed that nighttime sleep duration was an influencing factor of depression (*p* < 0.001); and using cognitive function as the independent variable and nighttime sleep duration and depression as the dependent variable, the results showed that depression was an influential factor of cognitive function (*p* < 0.001). The correlations among the study variables are presented in Table 4.

**Table 4:** Regression analyses of the effects of nighttime sleep duration and depression symptoms on cognitive functioning.

Dependent variable	Independent variable	*R*	*R*-sq	*F*	*p*	BEITA	*t*	*p*
Cognitive function	Nighttime sleep duration	0.086	0.007	31.082	<0.001	0.159	5.575	<0.001
Depression	Nighttime sleep duration	0.257	0.066	297.911	<0.001	−0.864	−17.260	<0.001
Cognitive function	Nighttime sleep duration	0.259	0.067	151.274	<0.001	0.038	1.336	0.182
	Depression					−0.139	−16.416	<0.001

**Figure 2:**
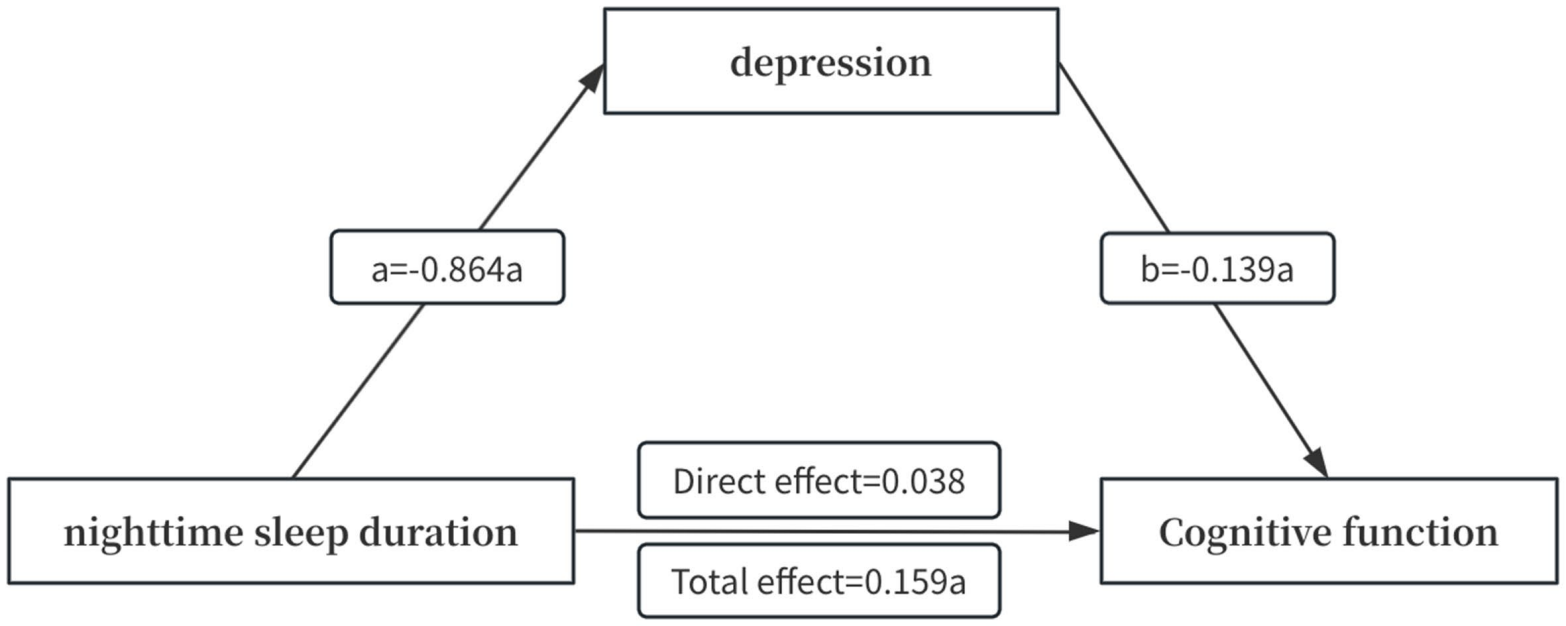
Modeling of the mediating effect of depression between nighttime sleep duration and cognitive functioning. a indicates *p* < 0.001.

PROCESS 4.4 was used to select model 4, and 5,000 self-sampling tests were conducted to test for mediating effects, which showed that the overall effect of nighttime sleep duration on cognitive functioning was 0.159, with a 95% CI (0.103, 0.214), and the direct effect was 0.038, with a 95% CI (−0.018, 0.094); and that the nighttime sleep duration through depression had an indirect effect of 0.120, 95% CI (0.100, 0.141). The results are shown in Table 5. The confidence intervals for the total and indirect effects did not contain 0, but the confidence interval for the direct effect did, indicating that nighttime sleep duration did not directly predict cognitive function, but rather predicted cognition through the mediating effect of depression, and that depression played a fully mediating role in the relationship between nighttime sleep duration and cognitive function. The path diagram of the mediating role is shown in Figure 2.

**Table 5:** Mediating effects of depression between nighttime sleep duration and cognitive functioning.

Path	Effect	Boot SE	95%CI		Proportion (%)
Total effect	0.159	0.028	0.103	0.214	
Direct effect	0.038	0.029	−0.018	0.094	24%
Indirect effect	0.120	0.011	0.100	0.141	76%

## Discussion

This study revealed significant associations between nighttime sleep duration, depressive symptoms, and cognitive function in middle-aged and older adult Chinese patients with multimorbidity. Notably, we found that nighttime sleep duration was positively associated with cognitive function, but the correlation coefficient was low. The results of grouping different sleep durations showed that there might be a “U” shaped relationship between nighttime sleep duration and cognitive function. Therefore, nighttime sleep duration should be reasonably controlled, and we should be alert to the adverse effects of too short or too long sleep.

In this study, the prevalence of cognitive dysfunction in middle-aged and older adult patients with multimorbidity was 35.7%, which was higher than the prevalence of older adults (≥60 years old, 25.27–27.69%) in previous studies (11), which may be related to the fact that the study population in this study was patients with multimorbidity. In addition, gender differences in the prevalence of cognitive dysfunction are controversial. The results of a meta-analysis (12) showed that the prevalence of cognitive impairment was higher in women than in men, which is consistent with the results of the present study, which may be due to the difference in socio-economic status between men and women. 38.2% of the participants reported a short duration of sleep at night, which is in line with the results of other studies of middle-aged and older adults (36.4%) (13). Depression symptoms were reported in 37.5% of middle-aged and older adults with multimorbidity, which is higher than other reports of depressive outcomes in older adults (13.0–27.5%) (14). One reason for this may be differences in the scales used to measure depression symptoms, and another may be demographic and regional differences.

The present study showed a negative correlation between the total depression score and the total cognitive functioning score, indicating that the more severe the depressive symptoms, the more pronounced the decline in cognitive functioning, which is in line with the results of a previous study (15, 16). A longitudinal study found that improving sleep in older adults may improve depression symptoms and delay cognitive decline. The reason for this may be that depression impairs cognitive functioning in individuals by impairing abilities such as attention and memory and reducing flexibility of thought and memory consolidation (17). Nighttime sleep duration was positively correlated with cognitive function, but the correlation coefficient was small. Therefore, we analyzed different nighttime sleep duration in groups, and the results showed that sleep duration was significantly negatively correlated with cognitive function in the short sleep duration group, while sleep duration was positively correlated with cognitive function in the long sleep duration group, suggesting that there may be a “U” shaped relationship between sleep duration and cognitive function, which is consistent with the results of other studies consistent with the results of other studies (18, 19). In the future, sleep-promoting interventions may significantly improve cognitive function in individuals with short sleep duration, whereas in individuals with long sleep duration, vigilance is needed against the potential adverse effects of excessive sleep on cognitive function. Nighttime sleep duration is negatively correlated with depression symptoms. People who do not get enough sleep at night usually suffer from sleep disorders such as insomnia, unhealthy diets, and other poor lifestyles, leading to an elevated risk of suffering from depression. In addition, a variety of chronic diseases may accelerate this process, and as the number of chronic diseases increases, the quality of life of patients decreases, leading to depression, anxiety, and other negative emotions. The results of a six-country cohort study (20) showed that older adults with multimorbidity had a significantly higher risk of depression than those without any disease. The pathogenesis of multiple chronic diseases is more complex than that of a single chronic disease, which is often accompanied by pain symptoms of varying degrees (21). For example, coronary artery disease combined with arthritis can lead to long-term physical discomfort or even inability to sleep at night, which seriously affects their quality of life, requires long-term treatment and medication management, and imposes a heavy financial burden on individuals and families, leading to the development of depression.

This study found a mediating effect between nighttime sleep duration, depression, and cognitive function in patients with multimorbidity. Depression played a fully mediating role in the relationship between nighttime sleep duration and cognitive function. The risk of depression in older adults rises progressively with age, exacerbated by progressively shorter sleep duration (22). Due to the abnormal secretion of melatonin and the disruption of sleep–wake cycle rhythms, older adults are prone to insomnia, early awakening, and even sleep disorders that induce anxiety and depression. Since sleep plays an important role in cognitive processes, especially memory consolidation and brain repair, sleep deprivation may accelerate cognitive deterioration in older adults by increasing their risk of depression. When older adults suffer from depression, they may actively reduce the frequency of social participation and social contact, leading to fewer opportunities for cognitive stimulation (23). At the same time, depressed mood may further reduce cognitive stimulation by affecting neurobiochemical processes and brain plasticity. As opportunities for cognitive stimulation decrease, older adults become less resistant to cognitive decline, leading to impairments in memory, attention, and other cognitive abilities.

## Limitations

There are some limitations of this study. First, nighttime sleep duration and prevalence in this study were self-reported by patients, which are subjective measurements and may be subject to recall bias and reporting bias, which may affect the results to some extent; second, some potential variables that may have an impact on cognitive functioning were not comprehensively included, such as medication use, sleep disorders, pain levels, and social engagement, which may have a nighttime sleep and cognitive functioning have a significant impact and should therefore be considered for inclusion in follow-up studies. Third, the relationship between different multimorbidity patterns and cognitive functioning has not been explored. In the future, different multimorbidity patterns can be analyzed using methods such as latent class or cluster analysis to explore their relationship with cognitive outcomes. Fourth, although we explored the relationship between nighttime sleep duration and cognitive function in subgroups, this relationship still does not explain causality. Future studies could use longitudinal mediation models with lagged variables and incorporate the concept of sleep health to analyze the causal relationship between sleep and depressive symptoms and cognitive functioning across different sleep perspectives (including duration, efficiency, satisfaction, and regularity).

## Conclusion

The state of cognitive functioning in middle-aged and older adult patients with multimorbidity in China is not optimistic, and there is an urgent need for effective interventions to prevent the development of cognitive dysfunction and mitigate its impact on the health of middle-aged and older adult people. Depression fully mediates the relationship between nocturnal sleep duration and cognitive function in middle-aged and older adult patients with multimorbidity. Based on this, when implementing cognitive function interventions for middle-aged and older adult patients with multimorbidity, relevant health departments and communities can use sleep management combined with psychological interventions to synergistically improve the cognitive dysfunction of middle-aged and older adult patients with multimorbidity, fully recognize the importance of mental health, and regularly organize and carry out mental health education activities, so as to increase middle-aged and older adult people’s knowledge and understanding of symptoms of depression, and to improve middle-aged and older adult people’s psychological tolerance; Popularize scientific knowledge of sleep hygiene, guide middle-aged and older adult people to develop good sleep habits, provide middle-aged and older adult people with appropriate sleep time, and promote cognitive health. At the same time, medical staff and nursing staff should pay close attention to the mental health status of middle-aged and older adult patients with multimorbidity, and provide timely psychological intervention and treatment for patients with depression tendency, so as to promote the overall improvement of the quality of life of middle-aged and older adult patients with multimorbidity.

## Data Availability

Publicly available datasets were analyzed in this study. This data can be found at: https://charls.charlsdata.com/.
